# Influence of Watershed Land Use on Contamination of the Sediments of Lake Victoria, Africa: A Case Study of Pesticides, Alkylphenols, and Antimicrobial Agents

**DOI:** 10.1021/acsenvironau.6c00060

**Published:** 2026-07-01

**Authors:** Merolyne Natuhwera, Liudmyla Khvalbota, Kenneth Arinaitwe, Krištof Urban, Edward Mubiru, Florence Nantaba, Peter Oswald, Zaccheus Shehu, George William Atwoki Nyakairu, Patrick Ssebugere, Ivan Špánik

**Affiliations:** † Department of Chemistry, 58588 Makerere University, P.O. Box 7062, Kampala 00000, Uganda; ‡ Institute of Analytical Chemistry, Faculty of Chemical and Food Technology, Slovak University of Technology in Bratislava, Radlinského 9, 812 37 Bratislava, Slovakia; § Johann Heinrich von Thünen-Institute of Fisheries Ecology, Herwigstraße 31, 27572 Bremerhaven, Germany; ∥ Department of Chemistry, Gombe State University, P.M.B. 127, Gombe 760214, Nigeria

**Keywords:** target analysis, pesticides, antimicrobial compounds, alkylphenols, sediment-dwelling organisms, ecological risks, Lake Victoria

## Abstract

Freshwater lakes are vulnerable to land-use changes that promote the input and accumulation of organic pollutants in sediments, posing risks to aquatic ecosystems. This study assessed the relationship between watershed land use and the occurrence of selected pesticides, antimicrobial agents, and alkylphenols in surface sediments of Lake Victoria in Africa. Surface sediment samples (*n* = 38) were collected in September 2019 across the Uganda, Kenya, and Tanzania sides of the lake. The samples were extracted using ultrasound-assisted extraction (UAE) and analyzed by gas chromatography–mass spectrometry (GC–MS). 16 out of 23 target compounds were detected, with pesticides showing low concentrations (<LOD to 0.662 μg/kg dry weight (d.w.)). Endrin-ketone and β-hexachlorocyclohexane (β-HCH) were the most frequently detected pesticides (frequency of appearance (FoA) at 42.1 and 28.9%, respectively). In contrast, alkylphenols and antimicrobial agents were ubiquitous, with 4-phenylphenol exhibiting the highest level (1,571.5 μg/kg d.w.). Spatially, elevated contaminant loads along the northern and northeastern shores of the lake coincided with catchments dominated by croplands and built-up areas, indicating the influence of agricultural runoff, urban wastewater, and riverine inputs on sediment contamination. Ecological risk assessment identified five priority compounds with risk scores >1 (4-*tert*-octylphenol, 2-phenylphenol, 4-phenylphenol, β-HCH, and 4-*n*-octylphenol) that pose potential aquatic ecotoxic risks to aquatic organisms. This study demonstrates that activities related to agriculture, industrialization, and urban development are the main drivers of sediment contamination in the lake, highlighting the need for targeted catchment management and monitoring.

## Graphical abstract

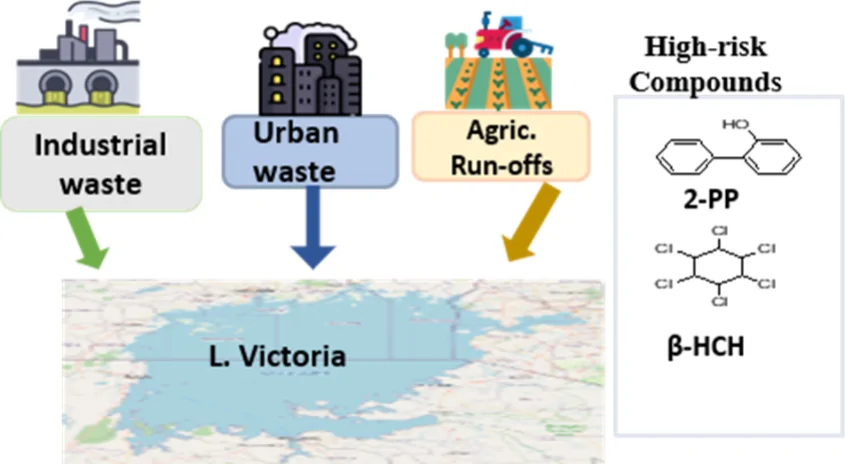

## 1 Introduction

Freshwater ecosystems worldwide are increasingly threatened by anthropogenic pressures associated with land-use and land-cover (LULC) change.^1^ Alterations in land use disrupt natural ecosystem services by modifying hydrological processes, sediment dynamics, and biogeochemical cycles. This, in turn, affects water quality and ecological integrity.^2,3^ In Sub-Saharan Africa, where agricultural expansion, urban growth, and industrialization are rapidly increasing, the impacts of LULC change on aquatic environments are more pronounced.^4,5^

The Lake Victoria basin, located in East Africa, is one of the most socio-economically and ecologically important freshwater systems in the world. The basin covers a catchment area of approximately 68,800 km^2^ and supports over 40 million people who depend on the lake for fisheries, domestic water supply, transportation, recreational activities, and agriculture. Agriculture remains the backbone of the regional economy, occupying large portions of the basin, alongside rapidly expanding urban centers and industrial activities.^6^ These land-use transitions have substantially increased diffuse and point-source pollution, resulting in the introduction of contaminants of emerging and established concern into the lake and its tributaries.^7^

Among the contaminants associated with intensive land use are alkylphenols, antimicrobial agents, and pesticides, which enter aquatic systems through agricultural runoff, wastewater discharges, and industrial effluents.^8,9^ Alkylphenols are degradation products of alkylphenol ethoxylates, a group of nonionic surfactants widely used in detergents, cleaning products, pesticides, industrial lubricants, and personal care products.^10^ Common examples include 4-*tert*-octylphenol, 4-*n*-octylphenol, and 4-*n*-nonylphenol. These compounds are well-known endocrine disruptors and have been linked to feminization of male fish, reduced sperm quality, impaired embryonic development, and disruption of thyroid hormone regulation in aquatic organisms.^11,12^ Their strong affinity for particulate matter promotes accumulation in sediments, which act as long-term sinks and potential secondary sources of contamination, thereby increasing ecological risks for benthic organisms.

Antimicrobial agents represent another important class of contaminants associated with urbanization and changing land-use patterns. These compounds are extensively used as preservatives and disinfectants in cosmetic, pharmaceutical, household, and healthcare products to prevent microbial growth.^13^ Common antimicrobial agents such as triclosan, triclocarban, 2-phenylphenol, 4-phenylphenol, and 1,4-dichlorobenzene are frequently detected in wastewater effluents due to their incomplete removal during conventional treatment processes.^14^ Once introduced into aquatic environments, they can persist, bioaccumulate, and exert toxic effects on aquatic biota by altering microbial communities, disrupting nutrient cycling, and inducing endocrine and developmental toxicity.^15,16^ Additionally, their widespread use has raised concerns about the promotion of antimicrobial resistance in environmental bacteria.

Pesticides are extensively applied in agricultural landscapes to control pests, weeds, and plant diseases, and their usage is strongly linked to land-use intensity and farming practices.^17^ In the Lake Victoria basin, intensive agriculture has contributed to the transport of pesticide residues into surface waters through runoff, erosion, and atmospheric deposition.^18^ Many pesticides exhibit high persistence and lipophilicity, favoring their accumulation in sediments, where they pose chronic risks to benthic organisms and higher trophic levels.^19^ Exposure of nontarget organisms such as fish and amphibians can disrupt food webs, reduce biodiversity, and alter ecosystem functioning.^20^ Furthermore, certain pesticide classes, particularly organochlorines, are associated with endocrine disruption, reproductive toxicity, neurotoxicity, and carcinogenic effects, raising concerns for both ecological and human health through bioaccumulation and trophic transfer.^21,22^

Previous studies have documented the occurrence of pesticides in Lake Victoria.^7,23–26^ However, these investigations did not cover all pesticide compounds and have limited consideration of land-use influences across the wider basin. In contrast, alkylphenols and antimicrobial agents remain poorly studied in the sediments of Lake Victoria, despite their extensive use in agricultural, industrial, and urban settings within the basin. Given the strong link between LULC patterns and contaminant inputs, a comprehensive, multiclass assessment is necessary to better understand how watershed land-use management influences sediment contamination. The conceptual basis of this study is that land-use and land-cover change determine the nature and intensity of contaminant sources, which are mobilized through hydrological pathways such as runoff, erosion, and wastewater discharge and ultimately accumulate in lake sediments depending on their physicochemical properties. Sediments, therefore, integrate spatial signals of watershed-scale anthropogenic activities and provide a useful matrix for assessing land-use-driven pollution.

To address the identified knowledge gaps, this study aimed at (i) determining the concentrations and spatial distribution of selected pesticides, antimicrobial agents, and alkylphenols in the sediments of Lake Victoria, thereby establishing a basin-wide contamination baseline; (ii) evaluating the ecological risks posed by these contaminants to sediment-dwelling organisms to identify priority pollutants of concern; and (iii) examining the relationship between land-use and land-cover (LULC) patterns and sediment contaminant distribution to elucidate the role of watershed activities as key drivers of pollution in the lake ecosystem.

## 2 Materials and Methods

### 2.1 Study Area

The study was conducted on Lake Victoria in Africa, which has a water volume of 2760 km^3^ and a total catchment area of 193,000 km^2^. The lake is distributed across Tanzania (49%), Kenya (6%), and Uganda (45%) (Figure 1). Stretching 412 km across the equator between latitudes 0°30′N and 3°12′S, and 355 km between longitudes 31°37′E and 34°53′E, the lake has an average depth of 40 m and a maximum depth of 80 m. The Lake Victoria basin is characterized by heterogeneous land-use patterns, including intensive agriculture, rapidly expanding urban centers, and localized industrial activities, which strongly control sediment and contaminant inputs to nearshore zones.

**Figure 1:**
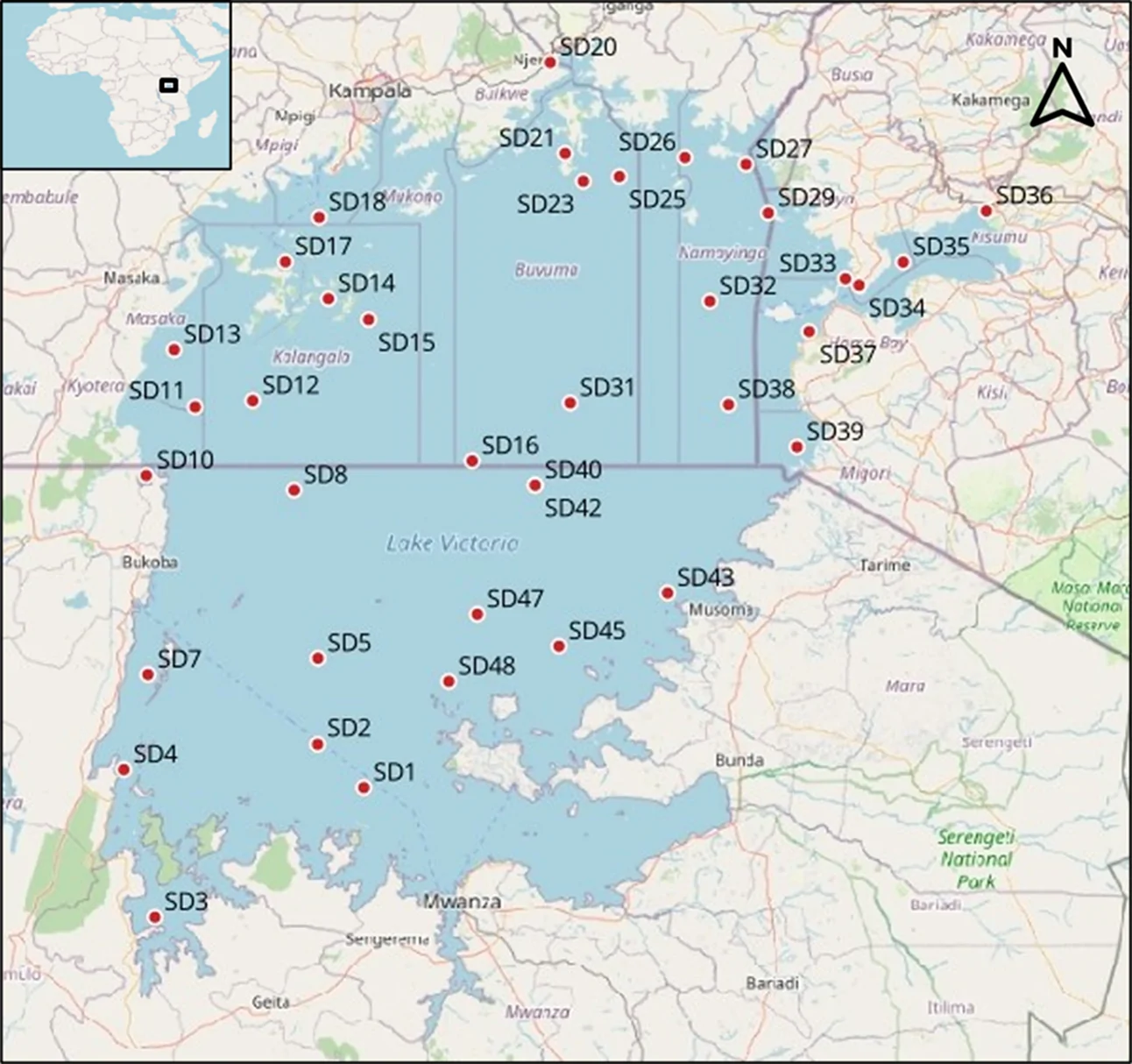
Geographic location of Lake Victoria showing the sampling sites.

### 2.2 Sampling

Surface sediment samples (*n* = 38, depth <10 cm) were collected from Lake Victoria in September 2019 using a grab sampler across three sides of the lake (Table S1 of the Supporting Information). Sampling sites were selected to represent major river inflows, urbanized shorelines, agriculturally influenced areas, and open-lake reference conditions across the three riparian countries, with technical assistance from the National Fisheries Resources Research Institute (NaFFIRRI). The collected samples were placed in polyethylene zip-lock bags, sealed, and uniquely labeled with permanent markers for identification. They were transported in cooling boxes packed with dry ice to the Institute of Analytical Chemistry, Faculty of Chemical and Food Technology, Slovak University of Technology in Bratislava. Upon arrival in the laboratory, the samples were freeze-dried.

### 2.3 Reagents and Standards

The reagents used in this study included acetonitrile (analytical grade), QuEChERS dSPE (900 mg anhydrous MgSO_4_ + 150 mg PSA + 15 mg GCB) from Phenomenex (Torrance, USA), and *n*-hexane.

Reference and internal standards for alkylphenols and pesticides were obtained from LGC Dr. Ehrenstorfer (Guildford, U.K.). Working standard solutions for both recovery tests and calibration were prepared by diluting portions of the stock standard solutions with methanol as the solvent. The standards were stored in a refrigerator at 4 °C.

### 2.4 Sample Extraction and Cleanup

Extraction was performed in accordance with the method reported by Łozowicka et al.^27^ Dry sediment samples (5 g) were placed in a 50 mL centrifuge tube. Purified water (10 mL) was added, vortexed for 1 min using a vortex (brand Heidolph Instruments, model Reax control, origin Schwabach, Germany), and allowed to stand for 10 min. Labeled internal standards (50 ng/g) were spiked and allowed to equilibrate at room temperature for 15 min. Acetonitrile (10 mL) was added to the centrifuge tube and vortexed for 7 min. The resultant mixture was ultrasonicated for 15 min using a sonicator (brand ELMA, model S 30 H Elmasonic, origin Singen, Germany), followed by centrifuging at 4000 rpm for 5 min using a centrifuge (brand Hettich, model Rotofix 32, origin Tuttlingen, Germany). The supernatant was then collected in an evaporation flask. The extraction process was repeated one more time in order to get pooled extracts.

The pooled extract (10 mL) was transferred into a centrifuge tube with dispersive-solid-phase extraction (QuEChERS d-SPE) (900 mg anhydrous MgSO_4_ + 150 mg PSA + 15 mg GCB). It was then vortexed for 1 min, followed by centrifuging at 4000 rpm for 5 min. The supernatant was evaporated to dryness using a gentle stream of nitrogen gas (>99.999% purity). The extract was then reconstituted in 1 mL of *n*-hexane before transferring it into a 2 mL vial for GC–MS analysis.

### 2.5 GC–MS Analysis

Chromatographic analysis was performed using an Agilent 7890A gas chromatograph coupled with a 5975C mass selective detector (MSD). Clean sediment extracts were introduced into the injector operating in the solvent vent mode. 10 μL was injected into the inlet with an initial temperature of 70 °C and then programmed at a rate of 600 °C/min until 290 °C and held at that temperature during the rest of the analysis. Separation was carried out in a 30 m × 250 μm × 0.25 μm HP-5 MS capillary column (Agilent). The carrier gas was helium at a constant flow rate of 1 mL/min, and the oven temperature was programmed at 50 °C (held for 2 min) to 200 °C at a rate of 25 °C/min, from 200 °C (held for 9.4 min) to 220 °C at a rate of 20 °C/min, from 220 °C (held for 1.4 min) to 300 °C (held for 0.7 min and equilibrated for 0.25 min). The MSD conditions included the following: the ion source temperature 250 °C, and the quadrupole temperature 150 °C; ionization energy 70 eV; operating in selected ion monitoring mode (SIM); selected ions monitored have been included in Table S2 of the Supporting Information. In order to obtain the full mass spectra of the compounds, a full scan analysis was performed. Compound identification was performed using the NIST2020 database. Certified reference standards were prepared in a concentration range of 0.5–50 ppb. Calibration curves were constructed in the range of 0.5–50 ppb. Quantification was performed using the external standard calibration method. The final concentration in the sediment was converted to μg/kg d.w.

### 2.6 Quality Control and Quality Assurance

Limits of detection (LOD) and limits of quantification (LOQ) were calculated based on the standard deviation (*S*_*y*_) of the responses and the slope (*S*) of the calibration curve as follows: LOD = 3­(*S_y_*/*S*) and LOQ = 10­(*S*_*y*_/*S*). Recovery tests were performed at two spiking levels, i.e., 1 mL of 0.5 ppm (100 μg/kg d.w.) and 1 mL of 1 ppm (200 μg/kg d.w.) of the standard mixture. For quality control and quality assurance, one procedural blank was analyzed in every batch of 10 sediment samples to check for cross-contamination. All analytes showed acceptable linearity (*R* ^2^ > 0.95), except 4-phenylphenol (*R* ^2^ = 0.94), which was retained given its environmental relevance. The equations for the calibration curves and their corresponding *R* ^2^ values are presented in Table S3 of the Supporting Information. The LODs ranged from 0.001 μg/kg d.w. (for trifluralin) to 1.93 μg/kg d.w. (for 4-phenylphenol), while the LOQs varied from 0.003 to 6.43 μg/kg d.w. for the same compounds. These results ensure the method’s sensitivity in detecting trace levels of alkylphenols in environmental samples.

### 2.7 Statistical Data Analysis

Descriptive statistics were calculated using Python (NumPy and Pandas libraries) to summarize each compound’s distribution. To assess spatial variability and differences in contaminant concentrations across the three riparian countries, various statistical tools were used. The Shapiro–Wilk test was conducted in Minitab statistical software (https://www.minitab.com/en-us/products/minitab/) to assess the normality of the alkylphenol data. A nonparametric Kruskal–Wallis test was later performed in Python (SciPy library) because our data set was not normally distributed to determine whether there were significant differences in analyte concentrations across the sampling points. Spatial distribution was analyzed and visualized using QGIS desktop 3.42.0 (https://www.qgis.org/project/visual-changelogs/visualchangelog342/).

Land-use and land-cover (LULC) classification of the Lake Victoria basin was developed using 2019 Sentinel-2 satellite imagery. A basin boundary shape-file was used to delineate the Lake Victoria basin as the area of interest within ArcGIS 10.8 GIS software. The Sentinel-2 land-cover data were extracted for the basin extent and subsequently reclassified into major LULC categories relevant to watershed-scale analysis. Lakes, rivers, and wetlands were grouped as water; agricultural areas as croplands; forested areas as forest; grasslands and savannahs as grassland; urban and built-up areas as built-up; and sparsely vegetated or exposed surfaces as bare areas. The resulting LULC map was used to characterize spatial land-use patterns across the Lake Victoria basin and to support the assessment of relationships between watershed land use and sediment contamination. Land-use patterns were used to support qualitative interpretation of spatial contamination trends rather than formal statistical correlation analysis. This approach enables the identification of dominant land-use drivers influencing contaminant accumulation in sediments at the basin scale.

### 2.8 Ecological Risk Assessment

Ecotoxicological risks posed by the target compounds were assessed following the method described in the previous studies.^28^ ^,^ ^29^ Briefly, risk quotients (RQs) were calculated from the ratio of measured environmental concentrations (MECs) to predicted no-effect concentrations (PNECs). The PNEC value is usually calculated as a ratio of ecotoxicological end point to assessment factor (AF) in environmental risk assessment (ERA) of chemicals.^30^ In this study, all PNEC values were obtained from the existing Norman ecotoxicological database (https://www.norman-network.com/nds/ecotox/lowestPnecsIndex.php). Three parameters were used for prioritization of compounds to be monitored in the lake’s basin in future studies, i.e., frequency of appearance (FoA), frequency of PNEC exceedance (FoE), and extent of PNEC exceedance (EoE). The FoA value represents the number of times the compound’s concentration was above the limit of detection, while FoE describes the frequency of sampling sites with concentrations exceeding PNEC values. The EoE value, which was calculated using (eq 1), indicates the magnitude of exceedance relative to toxicity thresholds.

$$\mathrm{EoE}=\frac{{\mathrm{MEC}}_{95}}{\mathrm{PNEC}}$$ 1

where MEC_95_ = 95th percentile of all MECsite values per compound.

Whenever an EoE value was in the range of 1–10, 10–100, 100–1000, and >1000, it was assigned 0.1, 0.2, 0.5, and 1 points, respectively. Risk scores were computed by adding up FoA, FoE, and EoE points. Using a scale of 0–1, compounds with a risk core >1 were considered for future monitoring studies in the Lake Victoria basin.

## 3 Results and Discussion

### 3.1 Concentrations (μg/kg d.w.) and Spatial Distribution of Pesticides in Sediments from Lake Victoria Relative to Land-Use Patterns

Pesticide contamination in Lake Victoria sediments showed clear spatial structuring associated with agriculturally dominated catchments. Out of 18 analyzed pesticides, 11 compounds were detected in at least one sample (Table S4), while 7 were not detected at all. Detected concentrations were generally low (<LOD to 0.662 μg/kg d.w.), which indicates limited accumulation in sediments. The most frequently detected compounds were β-HCH, endrin-ketone, and δ-HCH with frequencies of appearance (FoA) of 42.1, 28.9, and 28.9%, respectively. Endrin-ketone and β-HCH had the highest overall frequency of detection. The high detection frequency of β-HCH may be attributed to its high chemical stability and persistence in the environment, as it is the most thermodynamically stable isomer of HCH and resists microbial and photochemical degradation.^31^ β-HCH and HCB exceeded their environmental thresholds, i.e., PNEC = 0.001 and 0.0169 μg/kg d.w., respectively. The detection of pesticides like atrazine and legacy organochlorine isomers such as HCH isomers indicates that some pesticides are from ongoing agricultural practices and/or historic application.^26^

Despite their low concentrations, pesticides showed high spatial heterogeneity. Elevated levels were observed along the northeastern shoreline near Sia, Kisumu, Namayingo, and Homa Bay (Figure 2a). These areas are influenced by major river inflows draining intensively cultivated catchments such as Nzoia, Yala, and Sondu-Miriu.^32,33^ Therefore, the spatial coincidence between higher pesticide concentration and river mouth highlights the role of watershed-scale transport in delivering sediment-bound pesticides to the nearshore locations. Furthermore, comparison with land use and land cover (LULC) patterns (Figure 2b) shows that cropland (yellow) and built-up areas (red) are the dominant land-cover classes across the northeastern region, which reinforces the influence of agricultural activities on pesticide distribution. This implies that agricultural runoffs are the main sources of pesticides in the Lake Victoria basin.

**Figure 2:**
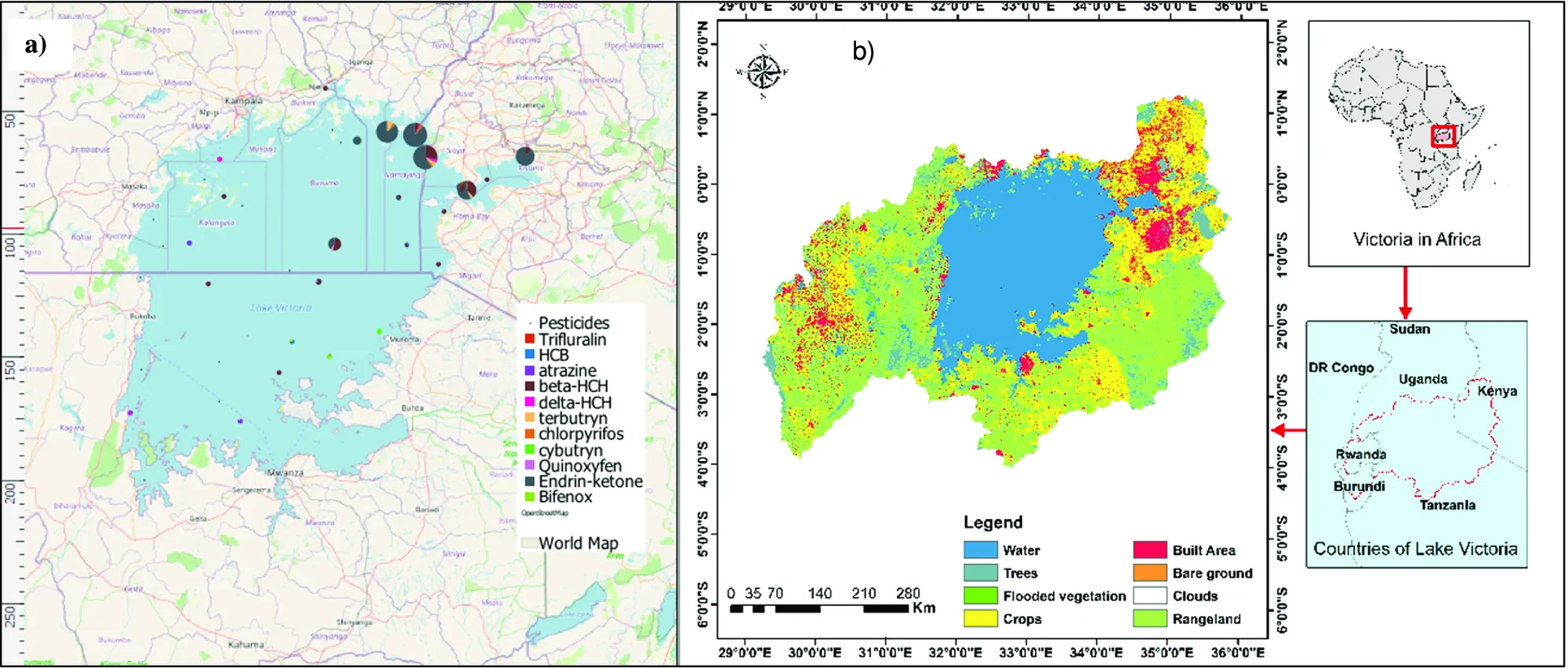
Map of the Lake Victoria basin showing (a) the spatial distribution of pesticides in sediments and (b) the land use classification.

This spatial land-use map correlates almost perfectly with hotspots of pesticide contamination. Because they are hydrophobic and particle-binding, these chemicals move adsorbed to eroded soil and accumulate in nearshore sediments, exactly where high concentrations appear, which demonstrates agro-hydrological coupling. While a full quantitative relationship was not performed, this co-occurrence suggests a land-use influence on sediment contamination patterns. However, further statistical analysis is required to confirm the strength of these relationships.

The observed levels of pesticides were in the same range as endrin OCPs reported in Lake Victoria sediments (n.d. to 2.89 μg/kg d.w.)^26^ but lower than those observed in the Ebro River basin in Spain (up to 112.18 μg/kg d.w.).^19^

### 3.2 Concentrations (μg/kg d.w.) and Spatial Distribution of Alkylphenols and Antimicrobial Agents in Sediments of Lake Victoria

Contrary to pesticides, which were detected at low frequencies, many alkylphenols and antimicrobial agents (2-phenylphenol, 4-phenylphenol, and 4-*tert*-octylphenol) had higher frequencies of detection (100%). The detected concentrations ranged from <LOD to 1571 μg/kg d.w., with 4-phenylphenol showing the overall maximum concentration (1571.5 μg/kg d.w.). The alkylphenols are known for their hydrophobic nature and affinity for organic matter, which explains their retention in sediments.^34^ Among the phenols, 2-phenylphenol and 4-phenylphenol were most abundant in sediments from the Kenyan side of the lake, with mean: 89.5 and 207.6 μg/kg d.w., respectively. Conversely, 4-*tert*-octylphenol was dominant in the sediments from Tanzania (mean: 122.5 μg/kg d.w.). Although 4-*n*-octylphenol and 4-*n*-nonylphenol were less frequently detected, their mean concentrations (185.5 and 2.5 μg/kg d.w., respectively) were highest in Tanzania (Table 1). All industrial chemicals exceeded their environmental thresholds in at least one of the samples, i.e., PNEC = 0.0340, 5.9110, 244.0668, 0.0034, and 0.1800 μg/kg d.w., for 4-*t*-OP, 2-phenylphenol, 4-phenylphenol, 4-*n*-octylphenol, and 4-*n*-nonylphenol, respectively.

**Table 1:** Concentrations of Alkylphenols in Sediments from Lake Victoria by Region (μg/kg d.w.)^a^

**compound**	**region**	**freq (%)**	mean ± SD	**min.**	**median**	**max.**
2-phenylphenol	Uganda	100	44.36 ± 40.0	5.92	45.50	96.40
	Kenya	100	89.50 ± 94.50	1.40	46.50	262.70
	Tanzania	100	78.00 ± 66.20	4.20	41.20	233.50
4-*tert*-octylphenol	Uganda	100	117.90 ± 125.90	16.90	78.90	422.30
	Kenya	100	60.60 ± 52.70	13.30	41.20	157.90
	Tanzania	100	122.50 ± 105.30	7.10	89.10	294.60
4-phenylphenol	Uganda	100	159.50 ± 161.50	6.70	123.70	577.70
	Kenya	100	207.60 ± 194.60	4.90	103.70	525.90
	Tanzania	100	196.80 ± 240.40	27.40	90.30	737.80
4-*n*-octylphenol	Uganda	17	73.63 ± 45.80	n.d.^b^		126.01
	Kenya	14	104.30 ± 104.30	n.d.		104.30
	Tanzania	15	185.50 ± 28.90	n.d.		214.40
4-*n*-nonylphenol	Uganda	11	1.75 ± 28.90	n.d.		2.50
	Kenya	0		n.d.		
	Tanzania	54	2.50 ± 2.90	n.d.		8.65

a Results are presented as mean **±** standard deviation (*n* = 18 for Uganda, 7 for Kenya, 13 for Tanzania).

b n.d.—non-detectable.

Despite these regional differences in concentration, a Kruskal–Wallis test showed no statistically significant variation in median concentrations across the three sides of the lake (*p* > 0.05). This could be attributed to overlapping land-use practices, including agriculture, urban development, and industrial activities. These shared activities contribute to similar types and levels of pollutants entering the lake from different regions. The Lake Victoria Environmental Management Program (LVEMP) study on pollutant loads reported 87 large towns in the Lake Victoria basin (51 in Kenya, 30 in Tanzania, 6 in Uganda) and 68 major industries (16 in Kenya, 34 in Tanzania, 18 in Uganda).^35^ These counts are based on data from 2002, and given the population growth and urban expansion over the past two decades, current numbers are likely higher. Both industrial and urban sites have been associated with high levels of alkylphenols in lakes in the USA and rivers in China.^36,37^ As seen in Figure 2b, there are many built-up areas (red) surrounding the lake, with most of them dominant in the northeastern region, eastern Uganda, and western Kenya. This spatial land-use map correlates with high concentrations of antimicrobial compounds: 2-phenylphenol, 4-phenylphenol, and an alkylphenol, 4-*t*-OP, observed in the northeastern region (Figure 3), but there are also other built-up areas surrounding the lake, which partly accounts for why high concentrations of similar compounds were observed in other areas of the lake.

**Figure 3:**
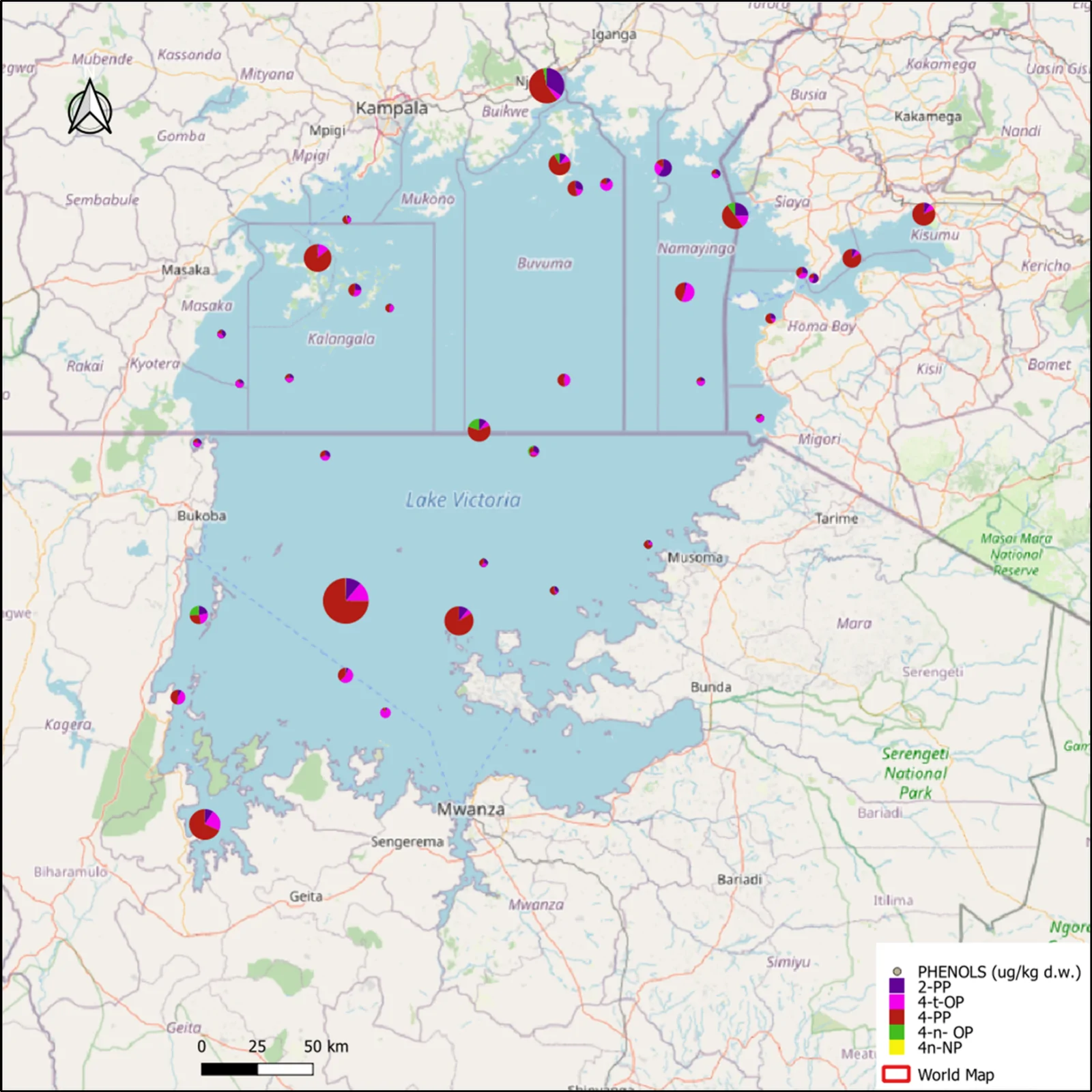
Spatial distribution of phenols (alkylphenols and antimicrobial agents) in sediments of Lake Victoria.

Since the compounds are hydrophobic, they bind to particles and get transported together with eroded soil accumulating in nearshore sediments, where concentrations are highest. However, wave action and transportation throughout the water column may influence the concentrations of similar compounds observed in the middle section of the lake. This was not evaluated in this study, and we recommend further studies to analyze these compounds in the water column.

Generally, the concentrations of alkylphenols in the sediments of this study were higher than those reported in Malaysia,^38^ Norway,^39^ and Minnesota lakes (USA)^40^ (Table 2). However, the levels were in the same range of data as those reported in the Great Lakes (USA and Canada), Ghanaian lakes, the Ebro River basin in Spain, and estuarine areas in Mumbai, India. Alkylphenols are widely used in industries and personal care products and therefore are expected to be particularly high in urbanized or industrialized areas. Compared to highly industrialized regions like the USA, China, and Spain, the Lake Victoria region (East Africa) is less industrialized, which explains the lower levels of alkylphenols observed in this study. However, the region is experiencing rapid urbanization with inadequate waste management systems, which could lead to higher releases of alkylphenols into the lake over time. This could result in their gradual accumulation in sediments, posing long-term ecological risks. Therefore, it is critical to implement better waste management practices and pollution control measures to protect the Lake Victoria ecosystem and mitigate potential future contamination.

**Table 2:** Concentrations of Alkylphenols and Antimicrobial Agents in Sediments of Lake Victoria^a^

water body	compounds	concentrations (μg/kg d.w.)
Lake Victoria, East Africa	2-PP, 4-*t*-OP, 4-PP, 4-*n*-OP 4-NP	<LOD to 1571.50 (this study)
lagoons: Fosu, Chemu, Korle; lake: Ashaiman Dam, rivers: Densu, Kakum, Ghana	4-NP, 4-*t*-OP	2022–6047 ^41^
Klang River Estuary, Malaysia	4-NP, 4-*t*-OP, E1, BPA	<LOD to 38.5 ^38^
Pearl River Delta, China	4-NP, E1, E2, BPA, 4-*t*-OP	<0.9 to 16,198 ^37^
Mumbai estuarine, India	4-NP, 4-*t*-OP	356.5 ^42^
Arctic Marine (Svalbard Islands), Norway	4-NP, precursors	0.3–21.0 ^39^
French river, France	NP, OP	14.2–95.2 ^43^
Minnesota lakes, USA	4-NP, 4-*t*-OP	up to 159 ^40^
Great Lakes, USA	4-NP, 4-*t*-OP	up to 37,000 ^36^
Ebro River Basin, Spain	NP, OP	1.11–5998.92 ^19^

a These levels have been converted to appropriate units, specifically from ng/g and μg/g to μg/kg, where applicable, to ensure consistency and enable meaningful comparisons across studies conducted in Lake Victoria and other global regions.

The concentration of phenols in Lake Victoria exhibited spatial heterogeneity (Figure 3). High concentrations of phenols are represented by larger pie charts, while smaller ones indicate lower levels. The lower levels were recorded in more isolated areas (western and central sections) of the lake. Contamination levels were highest in the northern and northeastern parts of the lake. These areas are closer to Kampala and Jinja in Uganda and Kisumu in Kenya. In addition, the northeastern region of the lake has about 4 in-flowing rivers. These could be acting as major conduits for pollutant loads by transporting polluted sediments, agricultural runoffs, sewage, and domestic and industrial effluents. According to Nyamweya, Desjardins, Sigurdsson, Tomasson, Taabu-Munyaho, Sitoki, and Stefansson,^32^ the hydrodynamic conditions in Lake Victoria vary considerably across the basin. While water exchange between the gulfs, such as Nyanza and the open lake, remains limited, there are stronger currents in the western and central regions of the lake. Consequently, these circulation patterns may influence the transport and accumulation of contaminants in some areas, thus the elevated phenol concentrations detected in the northeastern parts of the lake. With an average depth of approximately 40 m, Lake Victoria is considered a shallow lake. Moreover, the northern and western shores in Uganda are even shallower.^33^

It would therefore be expected that frequent sediment disturbance by waves and mixing in shallow waters would reduce phenol concentration in nearshore sediments. However, continuous inputs from urban effluents and river discharges may maintain elevated contaminant levels in these areas. High phenol concentrations were also observed in the southern part of the lake. In contrast to the northern and western regions, the southern basin contains deeper depositional zones that encourage the settling of fine particles and associated contaminants, thereby enhancing the accumulation of phenols in sediments. In addition, Lake Victoria exhibits two distinct annual hydrodynamic phases: a thermally stratified period from September to May and a mixing period from June to August.^32^ Since the sediments were collected during the stratified phase, the observed distribution and accumulation of contaminants in sediments may reflect the prevailing limnological conditions as a result of a warmer, oxygenated epilimnion overlying a cooler, oxygen-depleted hypolimnion.^44^

As such, phenols introduced into surface waters may not easily reach deeper sediments. Nevertheless, wind-driven mixing in shallow nearshore areas may alter sedimentation processes, resulting in variability in phenol accumulation and deposition. Reduced water circulation during stratification also lowers the potential for resuspension and redistribution, favoring the accumulation and retention of pollutants in the hypolimnion. Consequently, phenols deposited in deeper parts of the lake may be retained in sediments to a greater extent than in shallow, well-mixed areas. Moreover, oxygen-depleted conditions at depth may suppress microbial degradation processes, thereby increasing the persistence of certain phenolic compounds in the sediment environment. Conversely, areas located near major river inflows may exhibit localized enrichment of phenols as a result of continuous contaminant inputs and restricted dispersion during stratified conditions. In shallow nearshore zones, wind- and wave-induced disturbance can partially disrupt stratification, promoting the resuspension of sediment-associated compounds into the water column. Such processes may reduce the accumulation of phenols in surface sediments and could account for the comparatively lower concentrations observed at some nearshore sites relative to deeper depositional environments.

### 3.3 Prioritization of Detected Compounds for Future Monitoring

Risk-based prioritization in accordance with Slobodnik et al.^45^ identified only 8 out of 16 targeted compounds that exceeded the threshold criteria of FoE ≥ 1% (Table 3). 4-*tert*-Octylphenol, 4-*n*-nonylphenol, and hexachlorobenzene, which are compounds on the EU watchlist of dangerous environmental pollutants, are among the 8 compounds. 4-*tert*-Octylphenol and 4-*n*-octylphenol, which had concentrations more than 20,000 and 60,000 times higher than their PNEC, respectively, were of immediate concern due to high-risk margins. Compounds like 2-phenylphenol and β-hexachlorocyclohexane also had exceedances >150-fold, with high FoA of 100 and 42, respectively. These substances may pose chronic risks given their persistent occurrence across the basin. 4-*tert*-Octylphenol and 2-phenylphenol represent a dual concern, combining both high FoA (100%) and strong exceedance (20,000-fold and 150-fold, respectively).

**Table 3:** Risk Scores of the Compounds Based on Their PNEC, MEC, FoA, FoE, and EoE

compound	PNEC (μg/kg d.w.)	MEC (μg/kg d.w.)	95th MEC	FoA	FoE	EoE	points	risk score
4-*tert*-octylphenol	0.0340	777.7	404.4245	1	1	11,894.8400	1	3
2-phenylphenol	5.9110	944.4	309.9200	1	0.9475	52.4311	0.2	2.1474
4-phenylphenol	244.0668	1571.5	769.3600	1	0.3158	3.1523	0.1	1.4158
β-HCH	0.0010	0.393	0.3368	0.4211	0.4211	336.7500	0.5	1.3421
4-*n*-octylphenol	0.0034	214.41	199.9750	0.1579	0.1579	58,816.18	1	1.3158
4-*n*-nonylphenol	0.1800	8.65	6.8700	0.2368	0.2368	38.1667	0.2	0.6737
δ-HCH	6.4020	0.049	0.0315	0.2895	0	0.0049		0.2895
endrin-ketone	54.0560	0.662	0.6545	0.2895	0	0.0121		0.2895
hexachlorobenzene	0.0169	0.028	0.02785	0.0526	0.0526	1.6479	0.1	0.2053
terbutryn	2.3770	0.067	0.0658	0.1316	0	0.0277		0.1316
atrazine	0.2000	0.056	0.0538	0.0526	0.0263	0.2690		0.0789
chlorpyrifos	0.1146	0.032	0.0320	0.0789	0	0.2792		0.0789
cybutryn	0.0743	0.004	0.0040	0.0789	0	0.0538		0.0789
trifluralin	23.5360	0.024	0.0230	0.0526	0	0.0010		0.0526
bifenox	5.9470	0.013	0.0128	0.0526	0	0.0021		0.0526
quinoxyfen	27.0820	0.028	0.028	0.0263	0	0.0010		0.0263

Generally, only 5 compounds had a risk score >1, i.e., 4-*tert*-octylphenol, 2-phenylphenol, 4-phenylphenol, β-hexachlorocyclohexane, and 4-*n*-octylphenol, and were selected as priority compounds in this study (Table 3). 4-*tert*-Octylphenol was prioritized first, exceeding the established EQS PNEC in 100% of the sediment samples. The compound is an alkylphenol, a degradation product of nonionic surfactants, alkylphenol polyethoxylates, and a raw material for a number of industrial applications.^46^ It has a risk score of 3, and many studies in literature have reported its occurrence.^19,37–39^ This makes it a priority chemical of concern that requires the attention of regulators and researchers for further monitoring in the catchment.

The second prioritized compound was 2-phenylphenol with a risk score of 2.1474, followed by 4-phenylphenol, β-hexachlorocyclohexane, and 4-*n*-octylphenol, with risk scores of 1.4158, 1.3421, and 1.3158, respectively. Octyl phenols and β-hexachlorocyclohexane have also been reported with concern in the Ebro River catchment in Spain.^47^ The predominance of alkylphenols among the highest-risk compounds suggests a potential link to widespread usage in agricultural, industrial, and domestic applications within the basin, which are associated with land-use activities such as agriculture and urban development. Consequently, these compounds may represent priority pollutants in the sediments of Lake Victoria and are potential indicators of anthropogenic influence on the sediment quality. Based on this finding, these compounds could potentially pose a high risk to sediment-dwelling organisms and may be good candidates for Lake Victoria basin-specific pollutants. Alkylphenols accounted for four of the five highest-risk compounds, underscoring their disproportionate contribution to sediment ecological risk.

### 3.4 Limitations of the Study

Land-use analysis was based on the spatial overlay of raster data sets without direct statistical coupling to contaminant concentrations. Therefore, the observed relationships between land-use patterns and sediment contamination should be interpreted as associations rather than causal relationships (is nonconfirmatory but rather suggestive of plausible causation). Future studies integrating high-resolution land-use data with quantitative statistical approaches are recommended to better constrain the contribution of different land-use types to sediment pollution.

Furthermore, sampling was conducted during a single period (September, stratification phase), which may influence the observed distribution of contaminants and limit the ability to capture seasonal variability in Lake Victoria. Therefore, the results should be interpreted as a temporal snapshot rather than a full annual representation of contaminant dynamics.

## 4 Conclusion

This basin-wide sediment survey shows that while most pesticides occurred less frequently and at low concentrations (<LOD to 0.662 μg/kg d.w.), several alkylphenols and antimicrobial agents were ubiquitous, reaching up to 1571.5 μg/kg d.w. for 4-phenylphenol. Higher loads clustered along the northern and northeastern shores, aligning with urban wastewater and agriculturally influenced inflows. Ecological risk prioritization showed that only 4-*tert*-octylphenol, 2-phenyl phenol, 4-phenylphenol, β-HCH, and 4-*n*-octylphenol, with risk-scores >1, pose a potential threat to lake organisms. Therefore, they should be considered priority candidates for management and follow-up monitoring in the sediments of Lake Victoria. These results highlight the continuing relevance of legacy organochlorines alongside industrial chemicals. Strengthened wastewater control, catchment management, and periodic sediment surveillance focused on the identified priority substances are recommended. However, the lack of quantitative linkage between land-use and contaminant concentrations, together with the restriction to a single sampling period, limits causal interpretation and the assessment of seasonal variability. Therefore, future studies should incorporate quantitative approaches to strengthen source attribution and include multiseasonal sampling to better capture temporal dynamics.

## Supplementary Material



## Notes

Sampling details, analytical parameters, calibration data, and compound occurrence (Tables S1–S4) (PDF)
