# Supplementary material for: Influence of Watershed Land Use on Contamination of the Sediments of Lake Victoria, Africa: A Case Study of Pesticides, Alkylphenols, and Antimicrobial Agents
Source: ACS Environ Au. 2026 Jul 1;6(5):841–50. doi: 10.1021/acsenvironau.6c00060 (PMC13602339; doi:10.1021/acsenvironau.6c00060)
Supplement: Supplementary file 1 [file vg6c00060_si_001.pdf]

Influence of watershed land-use on contamination of the sediments of Lake Victoria-Africa: A case study of pesticides, alkylphenols, and antimicrobial agents

Merolyne Natuhwera [a,b], Liudmyla Khvalbota [b], Kenneth Arinaitwe [c] , Krištof Urban [b], Edward Mubiru [a], Florence Nantaba [a], Peter Oswald [b], Zaccheus Shehu [d], George William Atwoki Nyakairu [a], Patrick Ssebugere [a] , Ivan Špánik [b*]

[a]*Department of Chemistry, Makerere University, P.O. Box 7062, Kampala, Uganda*

[b]*Institute of Analytical Chemistry, Faculty of Chemical and Food Technology, Slovak University of Technology in Bratislava, Radlinského 9, 812 37 Bratislava, Slovakia*

[c]*Johann Heinrich von Thünen-Institute of Fisheries Ecology, Herwigstraße 31, 27572, Bremerhaven, Germany*

[d]*Department of Chemistry, Gombe State University, P.M.B 127, Gombe, Nigeria.*

Table S1: Coordinates of the sampling sites.

Sample code	Sampling site	Latitude (degrees)	Longitude (degrees)	Country
SD1	East Chelenche	-2.12527	32.55482	Tanzania
SD2	North Soswa	-1.97383	32.39332	Tanzania
SD3	Emin Pasha	-2.57813	31.82421	Tanzania
SD4	Chato	-2.06221	31.71558	Tanzania
SD5	Mazinga Island	-1.67308	32.39442	Tanzania
SD7	Norh Off Godziba	-1.73009	31.79988	Tanzania
SD8	Bumbire Channel	1.08512	32.3111	Tanzania
SD10	Rubafu Bay	-1.03412	31.79385	Tanzania
SD11	Off Sango Bay	-0.79452	31.96495	Uganda
SD12	West Lujabwa	-0.77243	32.16622	Uganda
SD13	Kagegi Gulf	-0.59436	31.89167	Uganda
SD14	Mbuguwe Island	-0.41595	32.43102	Uganda
SD15	Off Bukasa Island	-0.48911	32.57164	Uganda
SD16	Mid Lake	-0.98296	32.9337	Uganda
SD17	Utoboka Channel	-0.28681	32.28053	Uganda
SD18	Salysbury Channel	-0.13162	32.39882	Uganda
SD19	Off Murchison Bay	0.031378	32.61405	Uganda
SD20	Napoleon Gulf	0.410089	33.20661	Uganda
SD21	North Off Bugaiya	0.092178	33.25885	Uganda
SD23	East Bugaiya	-0.00528	33.32238	Uganda
SD24	Nambaga	0.160812	33.48018	Uganda
SD25	Off Luvia	0.010978	33.44842	Uganda
SD26	Off Sigulu	0.077705	33.67781	Uganda
SD27	Off Port Victoria	0.054032	33.89144	Uganda
SD28	Komogwe Island	-0.23817	33.90341	Uganda
SD29	Mageta Island	-0.11625	33.96914	Kenya
SD31	West Off Ugingo	-0.78015	33.2765	Uganda
SD32	Off Mfangano	-0.42483	33.76507	Uganda
SD33	Ngodhe	-0.34622	34.23906	Kenya
SD34	Naya Bay	-0.36875	34.28659	Kenya
SD35	Asembo Bay	-0.28796	34.44101	Kenya
SD36	Kisumu Bay	-0.10913	34.73172	Kenya
SD37	Off Takawiri	-0.53107	34.11203	Kenya
SD38	Off Migingo	-0.78628	33.83011	Uganda
SD39	Off Kuja River	-0.93434	34.06971	Kenya
SD40	South off Migingo	-0.93978	33.74053	Uganda
SD42	West Off Godziba	-1.06804	33.15384	Tanzania
SD43	Igenge Point	-1.44495	33.61717	Tanzania
SD45	North Irugua	-1.63091	33.23727	Tanzania
SD47	North Off Ukara	-1.51916	32.95166	Tanzania
SD48	Off Gana Island	-1.75381	32.85203	Tanzania

Table S2: Retention times and mass ions of monitored compounds.

No.	*Retention time, t_R* [min]	Compound	Abbreviation	CAS	*M* [g/mol]	SIM	ions
1	7.190	Dichlorvos	Dchl	62-73-7	220.98	109	185, 145
2	9.862	Trifluralin	Trf	1582-09-8	335.28	306	264, 290
3	10.332	α-hexachlorocyclohexane	aHCH	319-84-6	290.83	183	219, 111
4	10.524	Hexachlorobenzene	HCB	118-74-1	284.80	284	286, 249
5	10.627	Atrazine	Atr	1912-24-9	215.68	200	215, 173
6	10.821	β-hexachlorocyclohexane	bHCH	319-85-7	290.83	181	219, 109
7	11.494	δ-hexachlorocyclohexane	dHCH	319-86-8	290.83	181	219, 109
8	12.850	Alachlor	Ala	15972-60-8	269.77	160	188, 146
9	13.399	Terbutryn	Ter	886-50-0	241.36	226	185, 241
10	14.417	Chlorpyrifos	Chlp	2921-88-2	350.59	197	97, 314
11	16.156	Heptachlor epoxide (B, cis-, exo-)	HepE	1024-57-3	359.57	353	355, 81
12	16.268	Cybutryne (Irgarol)-D9		1189926-01-9	262.42	262	183
13	16.509	Cybutryne (Irgarol)*	Cyb	28159-98-0	253.37	182	253, 238
14	16.509	Chlorfenvinphos	Chlf	470-90-6	389.30	267	269, 323
15	20.186	Endrin	Edr	72-20-8	380.90	81	263, 281
16	21.002	Aclonifen	Acl	74070-46-5	264.67	264	183, 212
17	22.125	Quinoxyfen	Qnx	124495-18-7	308.14	237	307, 309
18	23.581	Endrin-ketone	EdrK	53494-70-5	380.90	67	317, 315
19	24.073	Methoxychlor	Mtch	72-43-5	345.60	227	228, 152
20	24.348	Bifenox	Bif	42576-02-3	342.10	341	343, 310
21	27.366	Cypermethrin 1	Cyp	52315-07-8	416.30	163	181, 165
22	7.998	4-*tert*-Octylphenol	4tO	140-66-9	206.33	279	135, 107, 206
23	9.305	4-*n*-Octylphenol	4nO	1806-26-4	206.32	280	107, 108, 206
24	10.407	4-*n*-Nonylphenol	4nN	104-40-5	220.35	317	107,220, 108

Table S3: Calibration curves, LODs, and LOQs for alkylphenols, antimicrobial agents, and pesticides.

Compound	R^2	Calibration Equation	LOD (µg/kg d.w)	LOQ (µg/kg d.w)
4-Phenylphenol	0.9394	Y = 33.045x – 60	1.9288	6.4300
2-phenylphenol	0.9635	Y =37.109x + 45	1.3274	4.4240
4-n-nonylphenol	0.9991	Y = 27603x – 1120	0.0609	0.2032
4-n-octylphenol	0.9992	Y = 26927x – 3534	0.0584	0.1946
4-tert-octylphenol	0.9823	Y = 21211x – 4651	0.2786	0.9284
Dichlorvos	0.9933	Y =13063368x -289	0.0016	0.0052
Trifluralin	0.9944	Y = 6237929x - 1508	0.0010	0.0030
Hexachlorobenzene	0.9971	Y= 10836040x -1134	0.0018	0.0056
Atrazine	0.9975	Y= 8363326x - 1661	0.0036	0.0116
β-hexachlorocyclohexane	0.9956	Y= 3194702x - 646.4	0.0340	0.1130
δ-hexachlorocyclohexane	0.9943	Y = 3060571x-717	0.0174	0.0576
Alachlor	0.9973	Y= 5086078x-851	0.0126	0.0422
Terbutryn	0.9897	Y=6165914x-1948	0.0072	0.0240
Chlorpyrifos	0.9943	Y= 3430651x-791	0.0250	0.0834
Cybutryn	0.9837	Y=5869273x-2050	0.0106	0.0352
Chlorphenvinfos	0.9869	Y=4817235x-1672	0.0190	0.0630
Endrin	0.9740	Y= 603345x + 20	0.1402	0.4674
Aclonifen	0.9780	Y = 1836574x-802	0.0194	0.0642
Quinoxyfen	0.9899	Y= 10618868x-1359	0.0096	0.0318
Endrin-ketone	0.9875	Y = 1988094x-794	0.0910	0.3032
Methoxychlor	0.9825	Y= 15690327x-5635	0.0030	0.0100
Bifenox	0.9715	Y= 1390572x-991	0.0506	0.1682
Cypermethrin	0.9930	Y= 3575013x+3531	0.0770	0.2566

Table S4: Concentrations of pesticides in sediments from Lake Victoria (µg/kg d.w.).

	**Count**	**Mean**	**STD**	**Min**	**25%**	**50%**	**75%**	**Max**
Trifl	3	0.013	0.012	0.000	0.007	0.014	0.019	0.024
HCB	2	0.027	0.002	0.025	0.026	0.027	0.027	0.028
Atr	3	0.032	0.025	0.006	0.020	0.034	0.045	0.056
β – HCH	16	0.144	0.122	0.014	0.041	0.113	0.255	0.393
δ-HCH	11	0.011	0.014	0.001	0.002	0.008	0.013	0.049
Terb	5	0.047	0.021	0.017	0.032	0.058	0.061	0.067
Chlor	3	0.029	0.005	0.024	0.028	0.032	0.032	0.032
Cyb	3	0.003	0.001	0.002	0.003	0.004	0.004	0.004
End 1	4	0.006	0.005	0.003	0.003	0.004	0.007	0.013
Qnx	1	0.028		0.028	0.028	0.028	0.028	0.028
End-K	11	0.281	0.255	0.014	0.061	0.190	0.471	0.662
Bif	2	0.011	0.004	0.008	0.009	0.011	0.012	0.013
